# The prevalence of gram-negative bacteria with difficult-to-treat resistance and utilization of novel β-lactam antibiotics in the southeastern United States

**DOI:** 10.1017/ash.2024.26

**Published:** 2024-03-18

**Authors:** Y. Vivian Tsai, P. Brandon Bookstaver, Joseph Kohn, Julie Ann Justo, Darrell Childress, Stefanie Stramel, Douglas Slain, Patrick Tu, Mary Joyce B. Wingler, Bruce M. Jones, Daniel T. Anderson, Megan M. Seddon, David A. Cretella, Joshua Eudy, Hana Winders, Kayla Antosz, Pamela Bailey, Majdi N. Al-Hasan

**Affiliations:** 1 Prisma Health-Midlands, Columbia, SC, USA; 2 Department of Clinical Pharmacy and Outcomes Sciences, University of South Carolina College of Pharmacy, Columbia, SC, USA; 3 Department of Internal Medicine, University of South Carolina School of Medicine, Columbia, SC, USA; 4 East Alabama Medical Center, Opelika, AL, USA; 5 Department of Pharmacy, Memorial Hermann Memorial City Medical Center, Houston, TX, USA; 6 Department of Clinical Pharmacy and Section of Infectious Diseases, West Virginia University, Morgantown, WV, USA; 7 Department of Pharmacy, Charlie Norwood VA Medical Center, Augusta, GA, USA; 8 Department of Antimicrobial Stewardship, University of Mississippi Medical Center, Jackson, MS, USA; 9 St. Joseph’s/Candler Health System, Savannah, GA, USA; 10 Department of Pharmacy, Augusta University Medical Center, Augusta, GA, USA; 11 Sarasota Memorial Health Care System, Sarasota, FL, USA

## Abstract

**Objective:**

To evaluate temporal trends in the prevalence of gram-negative bacteria (GNB) with difficult-to-treat resistance (DTR) in the southeastern United States. Secondary objective was to examine the use of novel β-lactams for GNB with DTR by both antimicrobial use (AU) and a novel metric of adjusted AU by microbiological burden (am-AU).

**Design:**

Retrospective, multicenter, cohort.

**Setting:**

Ten hospitals in the southeastern United States.

**Methods:**

GNB with DTR including Enterobacterales, *Pseudomonas aeruginosa*, and *Acinetobacter* spp. from 2015 to 2020 were tracked at each institution. Cumulative AU of novel β-lactams including ceftolozane/tazobactam, ceftazidime/avibactam, meropenem/vaborbactam, imipenem/cilastatin/relebactam, and cefiderocol in days of therapy (DOT) per 1,000 patient-days was calculated. Linear regression was utilized to examine temporal trends in the prevalence of GNB with DTR and cumulative AU of novel β-lactams.

**Results:**

The overall prevalence of GNB with DTR was 0.85% (1,223/143,638) with numerical increase from 0.77% to 1.00% between 2015 and 2020 (*P* = .06). There was a statistically significant increase in DTR Enterobacterales (0.11% to 0.28%, *P* = .023) and DTR *Acinetobacter* spp. (4.2% to 18.8%, *P* = .002). Cumulative AU of novel β-lactams was 1.91 ± 1.95 DOT per 1,000 patient-days. When comparing cumulative mean AU and am-AU, there was an increase from 1.91 to 2.36 DOT/1,000 patient-days, with more than half of the hospitals shifting in ranking after adjustment for microbiological burden.

**Conclusions:**

The overall prevalence of GNB with DTR and the use of novel β-lactams remain low. However, the uptrend in the use of novel β-lactams after adjusting for microbiological burden suggests a higher utilization relative to the prevalence of GNB with DTR.

## Introduction

The rise of antimicrobial resistance remains a pressing concern in medicine. There are an estimated 2.8 million antibiotic-resistant infections annually in the United States, causing more than 35,000 deaths.^1^ Extended-spectrum β-lactamases producing Enterobacterales (ESBL-E), carbapenem-resistant Enterobacterales (CRE), *Pseudomonas aeruginosa* with difficult-to-treat resistance (DTR *P. aeruginosa*), and multidrug-resistant (MDR) *Acinetobacter* spp. are some of the most worrisome gram-negative bacteria (GNB) contributing to the rise of antimicrobial resistance.^1,2^ As a result, multiple new, broad-spectrum β-lactam antibiotics, such as ceftolozane/tazobactam, ceftazidime/avibactam, imipenem/cilastatin/relebactam, meropenem/vaborbactam, and cefiderocol, have been developed for the treatment of infections caused by these organisms.^3–5^ However, as the use of these novel β-lactam antibiotics increases, the need for benchmarking their utility by antibiotic stewardship programs (ASP) in relation to the prevalence of the GNB with DTR is warranted to ensure appropriate use and prevent further resistance.

Antimicrobial use (AU) is a direct measurement that quantifies antimicrobial administration within an institution. Defined daily dose or days of therapy (DOT) per patient admission, per patient-days (PD), and per days present are metrics employed by various institutions to capture AU data.^6^ AU metric is limited for assessment of appropriate AU as it does not account for differences in hospital characteristics and epidemiology.^7,8^ To provide a more balanced comparison of AU across hospitals, it has been proposed to adjust AU by microbiological burden (am-AU) to account for the potential need of broad-spectrum antibiotics at each location based on local microbiology data.^9^ This novel metric has been previously used to examine AU of anti-pseudomonal β-lactams, carbapenems, anti-methicillin-resistant *Staphylococcus aureus* (MRSA) agents, and anti-vancomycin-resistant *Enterococcus* (VRE) agents in 26 hospitals in the United States.^10^

The primary objective of this retrospective, multicenter cohort study was to examine the temporal trends in the prevalence of GNB with DTR and the use of novel β-lactam antibiotics in the southeastern United States. The secondary objective was to examine the impact of adjustment of AU based on the prevalence of GNB with DTR at each hospital.

## Methods

The prevalence of GNB with DTR and the AU of novel β-lactam antibiotics were determined at 10 hospitals in the Southeastern Research Group Endeavor-45 (SERGE-45) research network located throughout the southeastern United States between 2015 and 2020. The GNB with DTR evaluated in this study were Enterobacterales, *P. aeruginosa*, and *Acinetobacter* spp. DTR was defined as GNB that were non-susceptible to all of the following: ceftazidime or cefepime, meropenem or imipenem/cilastatin, ciprofloxacin or levofloxacin, and piperacillin/tazobactam (if tested and reported).^11^ The prevalence of GNB with DTR for each hospital was calculated utilizing antibiogram data for calendar years 2015 through 2020. The cumulative prevalence of GNB with DTR was the total GNB with DTR isolate count divided by the total gram-negative isolate count for each hospital. The prevalence of DTR Enterobacterales was the count of DTR Enterobacterales divided by the total Enterobacterales isolate count. The prevalence of DTR *P. aeruginosa* was the count of DTR *P. aeruginosa* divided by the total *P. aeruginosa* isolate count. The prevalence of DTR *Acinetobacter* spp. was the count of DTR *Acinetobacter* spp. divided by the total *Acinetobacter* spp. isolate count for each hospital. Linear regression analysis was used to examine a change in the temporal trends in the prevalence of GNB with DTR from 2015 to 2020.

Novel β-lactam antibiotics evaluated were ceftolozane/tazobactam, ceftazidime/avibactam, imipenem/cilastatin/relebactam, meropenem/vaborbactam, and cefiderocol. AU was defined as DOT per 1,000 patient-days present. AU was determined for composite of all novel β-lactams as well as each individual novel β-lactam antibiotic at each hospital during calendar years 2015–2020. Linear regression was utilized to examine the temporal trends in cumulative AU of novel β-lactam antibiotics from 2015 to 2020.

Adjusted AU by microbiological burden (am-AU) was calculated as described by Al-Hasan et al.^9^ where am-AU of new β-lactam antibiotics was the AU of β-lactam antibiotics divided by the prevalence of GNB with DTR at that hospital divided by the average prevalence of GNB with DTR across all hospitals included. Hospitals were ranked by AU and am-AU from lowest to highest based on cumulative novel β-lactam antibiotic use. The impact of adjustment by microbiological burden was assessed by the proportion of hospitals that had a change in ranking between AU and am-AU of novel β-lactam antibiotics.

RedCap version 11.4.4 software was used for data collection and management. Excel 2016 software (Microsoft, Redmond, WA) and JMP Pro version 13.0 software (SAS Institute, Cary, NC) were used for statistical analyses. *P* < .05 (two-sided) was considered statistically significant. The Institutional Review Board at the University of South Carolina approved the study. It determined that this research is not subject to the Protection of Human Subject Regulations in accordance with the Code of Federal due to the lack of use of patient-specific data.

## Results

Table 1 shows the characteristics of the 10 hospitals included in the study. Six hospitals had ≥500 bed size. Antimicrobial stewardship activities were conducted onsite in all hospitals. A total of 8 hospitals had novel β-lactam antibiotics included on formulary with ceftolozane/tazobactam and ceftazidime/avibactam being the most common. For all hospitals, novel β-lactam antibiotics were restricted for use with protection criteria and required prospective audit and feedback by infectious diseases or antimicrobial stewardship teams.

**Table 1.** Characteristics of the ten participating hospitals in the study

State	Bed size	Type of stewardship	Pharmacist stewardship FTEs	Automated antimicrobial susceptibility system	EHR system	Included antibiotics on formulary	Included antibiotics with protection criteria	Included antibiotics with prospective audit and feedback
AL	201–500	Onsite	1	MicroScan	Cerner	C/T, CZA, MEV, IMR, FDC	C/T, CZA, MEV, IMR, FDC	C/T, CZA, MEV, IMR, FDC
GA	≤200	Onsite	1	MicroScan	CPRS	C/T, CZA, FDC	C/T, CZA, FDC	C/T, CZA, IMR, FDC
GA	≥501	Onsite	1	VITEK 2	Meditech		C/T, CZA, MEV, IMR, FDC	C/T, CZA, MEV, IMR, FDC
GA	≥501	Onsite	1.5	VITEK 2	Cerner	C/T, CZA, FDC	C/T, CZA, MEV, IMR, FDC	C/T, CZA, MEV, IMR, FDC
TX	201–500	Onsite	1	MicroScan	Cerner		C/T, CZA, MEV, IMR, FDC	C/T, CZA, MEV, IMR, FDC
FL	≥501	Onsite	1.7	VITEK 2	Allscripts	C/T, CZA, MEV, IMR, FDC	C/T, CZA, MEV, IMR, FDC	C/T, CZA, MEV, IMR, FDC
WV	≥501	Onsite	4	VITEK 2	Epic	C/T, CZA, MEV	C/T, CZA, MEV, IMR, FDC	C/T, CZA, MEV, IMR, FDC
SC	≥501	Onsite	1	VITEK 2	Epic	C/T, CZA, MEV, IMR, FDC	C/T, CZA, MEV, IMR, FDC	C/T, CZA, MEV, IMR, FDC
SC	201–500	Onsite	0.5	VITEK 2	Epic	C/T, CZA, MEV, IMR, FDC	C/T, CZA, MEV, IMR, FDC	C/T, CZA, MEV, IMR, FDC
MS	≥501	Onsite	2	VITEK 2	Epic	C/T, CZA, MEV	C/T, CZA, MEV, IMR, FDC	C/T, CZA, MEV, IMR, FDC

Abbreviations: AL, Alabama; CPRS, Computerized Patient Record System; C/T, ceftolozane/tazobactam; CZA, ceftazidime/avibactam; EHR, electronic health record; FDC, cefiderocol; FL, Florida; FTE, full-time equivalent; GA, Georgia; IMR, imipenem/cilastatin/relebactam; MEV, meropenem/vaborbactam; MS, Mississippi; SC, South Carolina; TX, Texas; WV, West Virginia

There were a total of 143,638 isolates of GNB in the antibiograms of the 10 participating hospitals during the 6-year study period. Enterobacterales, *P. aeruginosa*, and *Acinetobacter* spp. contributed 122,911 (85.6%), 18,513 (12.9%), and 2,214 (1.5%) of all GNB in the study, respectively. The overall prevalence of GNB with DTR at the 10 hospitals was 0.85% (1,223/143,638) and ranged from 0.30% to 1.54% across the 10 hospitals. There was a numerical increase in the prevalence of GNB with DTR from 0.77% (136/17,772) to 1.00% (255/25,456) between 2015 and 2020 (*P* = .06).

The overall prevalence of DTR Enterobacterales, *P. aeruginosa*, and *Acinetobacter* spp. during the study period was 0.21% (264/122,911), 3.7% (692/18,513), and 12.1% (267/2,214), respectively. There was a relative variation in the prevalence of bacteria with DTR across the 10 hospitals. The prevalence of DTR Enterobacterales ranged from 0% to 0.47%, DTR *P. aeruginosa* from 1.2% to 7.5%, and DTR *Acinetobacter spp*. from 0% to 30.8%.

**Figure 1.**
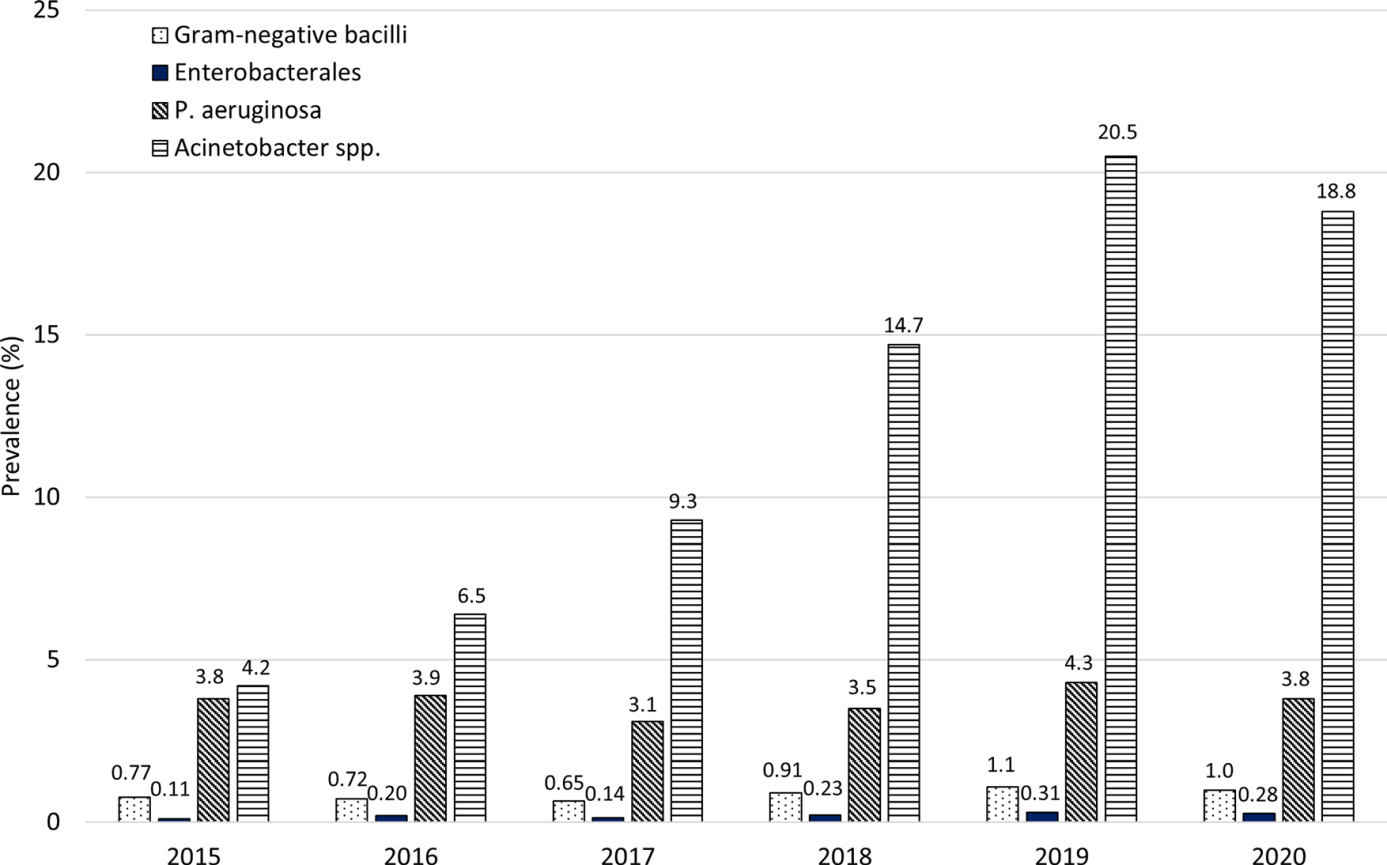
Prevalence of gram-negative bacilli with difficult-to-treat resistance from 2015 to 2020. *P* values for trends from 2015 to 2020: gram-negative bacilli (0.06), Enterobacterales (0.023), *P. aeruginosa* (0.69), *Acinetobacter* spp. (0.002).

The temporal trends in the prevalence of DTR isolates from 2015 to 2020 also differed across various GNB. While the prevalence of DTR. *P. aeruginosa* remained relatively stable, there was a significant uptrend in the prevalence of DTR Enterobacterales (0.11% [20/17,747] to 0.28% [60/21,799], *P* = .023) and *Acinetobacter* spp. (4.2% [16/383] to 18.8% [70/372], *P* = .002) as demonstrated in Figure 1.

**Figure 2.**
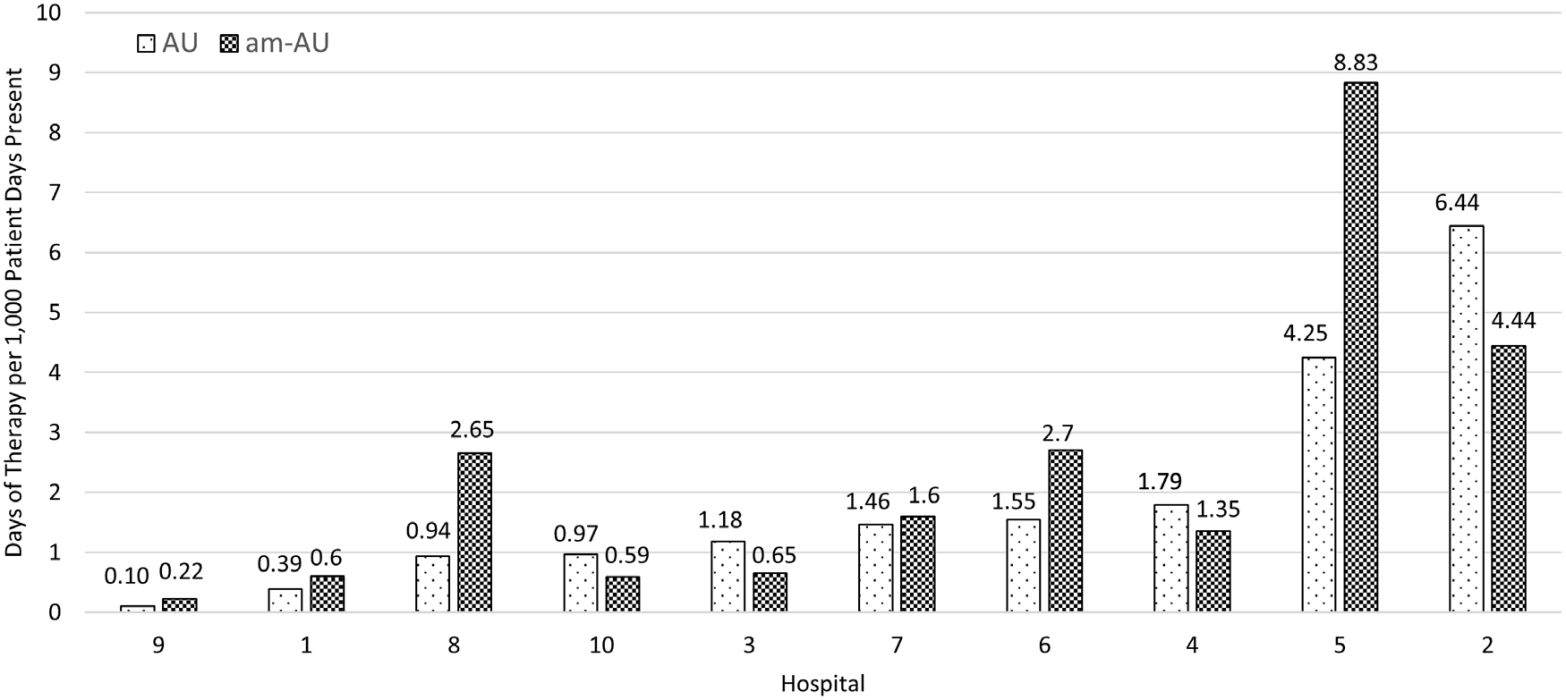
Cumulative antibiotic use versus adjusted antibiotic use of novel β-lactams by microbiological burden, ranked by hospital from lowest to highest, 2015–2020. *Note.* am-AU, adjusted antibiotic use; AU, antibiotic use.

The cumulative AU of novel β-lactam antibiotics at the 10 hospitals during the study period was 1.91 ± 1.95 DOT per 1,000 patient-days present with a range of 0.10–6.44 DOT per 1,000 patient-days present (Figure 2). Ceftolozane/tazobactam (0.94 ± 1.24 DOT per 1,000 patient-days present) and ceftazidime/avibactam (0.57 ± 0.58 DOT per 1,000 patient-days present) accounted for most AU (Table 2). The cumulative am-AU was 2.36 ± 2.62 DOT per 1,000 patient-days present with a range of 0.22–8.83 DOT per 1,000 patient-days present (Figure 2). Hospital ranking by AU had considerable changes after adjustment for microbiological burden. Eight of 10 hospitals had at least 1 position change in ranking, and 3 hospitals shifted at least 2 positions in ranking comparing AU to am-AU.

**Table 2.** Mean antibiotic use of novel β-lactams from 2015 to 2020

	FDA approval year	2015	2016	2017	2018	2019	2020	2015-2020^b^
Ceftolozane/tazobactam	2014	0.03 ± 0.08	0.18 ± 0.28	1.86 ± 3.57	1.11 ± 1.59	1.23 ± 0.93	1.24 ± 1.00	0.94 ± 1.24
Ceftazidime/avibactam	2015	*	0.07 ± 0.17	0.57 ± 0.66	0.65 ± 0.64	0.81 ± 0.80	0.73 ± 0.64	0.57 ± 0.58
Meropenem/vaborbactam	2017	*	*	*	0.07 ± 0.22	0.06 ± 0.18	0.11 ± 0.13	0.08 ± 1.8
Imipenem/cilastatin/ relebactam	2019	*	*	*	*	*	0.09 ± 0.24	0.09 ± 0.24
Cefiderocol	2019	*	*	*	*	*	0.03 ± 0.70	0.03 ± 0.70
**Cumulative** ^a^		0.03 ± 0.08	0.25 ± 0.42	2.54 ± 4.04	1.83 ± 1.91	2.10 ± 1.33	2.18 ± 1.28	1.91 ± 1.95

Antibiotic use is reported by mean days of therapy per 1,000 patient-day present ± standard deviation.

a Cumulative mean AU of all new β-lactam antibiotics by year.

b Mean antibiotic use during the entire study period for each novel β-lactam antibiotic.

* Use not reported.

## Discussion

The overall prevalence of GNB with DTR was <1% among participating hospitals located in the southeastern region of United States. This was in line with recent studies where the prevalence of DTR bacteria was reported to be 1% among 173 US hospitals between 2009 and 2013^11^ and 1.3% among 140 US hospitals between 2009 and 2015.^12^ The prevalence of DTR *P. aeruginosa* remained relatively steady overtime. This trend is crucial for maintaining appropriate use of novel β-lactam antibiotics and preserving their activity against DTR *P. aeruginosa*. On the other hand, the increasing prevalence of DTR Enterobacterales is worrisome as it could drive increased use of novel β-lactam antibiotics as this is the largest group of gram-negative pathogens that hospitals encounter. While the increased prevalence of DTR *Acinetobacter* spp. is alarming from an epidemiological standpoint, its impact on the future use of novel β-lactam antibiotics is unclear as most of the novel β-lactam antibiotics are not considered first-line agents for the treatment of DTR *Acinetobacter* spp. with the exception of cefiderocol. However, with the recent FDA approval of sulbactam-durlobactam, it is expected that the use of cefiderocol as the primary agent for the treatment of DTR *Acinetobacter* spp. will be minimized since cefiderocol monotherapy is not recommended per the Infectious Diseases Society of America (IDSA) guidance.^13^ In addition, although the prevalence of DTR *Acinetobacter* spp. approached 20% toward the end of the study period, *Acinetobacter* spp. represented only a small fraction of total GNB in this study.

The upward trends of cumulative AU of novel β-lactam antibiotics could be explained by the increasing availability of these agents since most of novel β-lactam antibiotics with the exception of ceftolozane/tazobactam were approved during the study period. Furthermore, the increased uptake of these agents can also be explained by the overall increase in the prevalence of GNB with DTR, especially in respect to Enterobacterales. Although the overall cumulative AU was relatively low, an increase was observed after adjusting for local microbiological burden. This finding suggests the increased use of novel β-lactam antibiotics relative to the observed prevalence of GNB with DTR. It is possible that the increase in utilization of novel β-lactam antibiotics may be a result of appropriate indication for disease states that may require longer duration (eg, osteomyelitis), which is not considered as part of the antimicrobial utilization evaluation in this study.

Benchmarking comparisons of antimicrobial resistance is now available through Centers for Disease Control and Prevention (CDC) Antimicrobial Use and Resistance (AUR) module. Specifically, the Standardized-Resistant Infection Ratio (SRIR) compares the rate of hospital-onset drug-resistant infection events to the national benchmark with adjustment for various facility level factors that contribute to the risk of antimicrobial resistance within each facility. Similar to the prevalence calculation used in the present study, SRIR is calculated by dividing the observed resistant infection by predicted resistant infection using national aggregated antimicrobial resistance data reported to the National Health Safety Network (NHSN). For GNB, SRIR can be generated for antibiotic-resistant Enterobacterales and *P. aeruginosa* from three different specimen sources including blood, urine, and lower respiratory tract, which could be beneficial to further elucidate the likelihood of colonization versus contamination for these extensively drug-resistant organisms. Unlike the definition used in the present study, the CDC AUR modules specifically characterize drug resistance to Enterobacterales into three different categories (eg, carbapenem-, extended-spectrum cephalosporin-, and fluoroquinolone-resistant) and *P. aeruginosa* as resistance to at least three of the six drug categories (eg, extended-spectrum cephalosporin, fluoroquinolones, aminoglycosides, carbapenems, piperacillin/tazobactam, and cefiderocol).^14^ The present study utilized DTR definition consistent with Kadri et al.’s^11^ study since the current clinical practice typically reserves the use of novel β-lactam antibiotics until all of the first-line agents have been exhausted as opposed to resistance to only a select few.^15^

The current metric of AU reporting for eligible healthcare facilities is based on the Standardized Antimicrobial Administration Ratio (SAAR). SAAR predicts AU using predictive models that adjust each specific antimicrobial agent category based on patient care location and facility type. Currently, novel β-lactam antibiotics are not included in SAAR antimicrobial agent categories for benchmarking purposes. However, as a supplement to the SAARs, AU of ceftolozane/tazobactam and ceftazidime/avibactam can be evaluated through SAAR rate table as part of the adult antibacterial agent group predominately used for extensively antibiotic-resistant bacteria. This rate table calculation is based on antimicrobial days per 1,000 days present along with the pooled mean rate and percentile distributions.^14^ The present study could not adjust AU based on patient location and facility type given the small sample size with relatively variable patient population. It remains unclear whether adjustments based on hospital characteristics or microbiological burden is more relevant.

To our knowledge, this is the first study to evaluate the prevalence of GNB with DTR in relation to AU of novel β-lactam antibiotics. With the observed increased prevalence of GNB with DTR, it is vital from the antimicrobial stewardship perspective to characterize the utilization of novel β-lactam antibiotics to ensure their appropriateness and minimize the risks for potential resistance. There are several limitations to this study. First, the inclusion of hospitals from the southeastern United States may not allow for generalization to hospitals in other areas as the pattern of resistance may differ. Second, the susceptibility testing of GNB with DTR is not standardized across institutions, which may influence the prevalence of GNB with DTR. Third, not all isolates reported in antibiogram are clinically relevant, which may influence the adjustment of AU based on microbiological burden. Fourth, the study did not evaluate alternative antibiotics (eg, polymyxins) that would have previously been utilized for DTR organisms. Finally, the study design does not allow assessment of the populations in which novel β-lactam antibiotics were unnecessarily used.

It is speculated that novel β-lactams may have been used empirically in patients with risk factors for or history of infections due to GNB with DTR. It is also possible that novel β-lactams may have been used for the treatment of infections due to carbapenem-resistant *P. aeruginosa* that were not necessarily DTR. Since carbapenemase production is not the dominant resistance mechanism, many of these carbapenem-resistant *P. aeruginosa* remain susceptible to other anti-pseudomonal β-lactams such as piperacillin/tazobactam and cefepime.^15^ Education of healthcare providers on the potential utility of other anti-pseudomonal β-lactams and fluoroquinolones for the treatment of infections due to carbapenem-resistant *P. aeruginosa* may reduce the utilization and costs associated with novel β-lactam antibiotics in many cases. The current study also demonstrates that adjusting AU by microbiological burden provides an objective method to compare AU of novel β-lactams across heterogeneous institutions with various hospital epidemiology. This adds to the previously demonstrated validity of this novel metric to assess AU of anti-pseudomonal β-lactams, carbapenems, anti-MRSA, and anti-VRE agents.^10^

In conclusion, although the overall prevalence of GNB with DTR and the cumulative use of novel β-lactam antibiotics remain relatively low, the uptrend in the prevalence of DTR isolates is concerning, especially in respect to the increase in DTR Enterobacterales. Furthermore, benchmarking the use of novel β-lactam antibiotics based on local microbiology resulted in a numerical increase in use suggesting potential higher utilization of novel β-lactam antibiotics in relation to observed prevalence of GNB with DTR.

## References

[1] CDC. Antibiotic Resistance Threats in the United States, 2019. Atlanta, GA: U.S. Department of Health and Human Services, CDC; 2019.

[2] WHO (2017). WHO publishes list of bacteria for which new antibiotics are urgently needed. World Health Organization. https://www.who.int/news/item/27-02-2017-whopublishes-list-of-bacteria-fo-which-new-antibiotics-are-urgenttly-needed.

[3] Ho S , Nguyen L , Trinh T , et al. Recognizing and overcoming resistance to new beta-lactam/beta-lactamases inhibitor combination. Curr Infect Dis Resp 2009;21:39. 10.1007/s11908-019-0690-9 31501948

[4] Zhanel GG , Lawrence CK , Adam H , et al. Imipenem-relebactam and meropenem-vaborbactam: two novel carbapenem-B-lactamase inhibitor combinations. Drugs 2018;78:65–98. 29230684 10.1007/s40265-017-0851-9

[5] Taheri Y , Jokovic N , Vitorovic J , et al. The burden of the serious and difficult-to-treat infections and a new antibiotic available: cefiderocol. Front Phrmacol 2021;11:578823. 10.3389/fphar.2020.578823 PMC7898678 33628170

[6] Barlam TD , Cosgrove SE , Abbo LM , et al. Implementing an antibiotic stewardship program: guidelines by the infectious diseases society of America and society for healthcare epidemiology of America. Clin Infect Dis 2016;62:e51–e77. 27080992 10.1093/cid/ciw118 PMC5006285

[7] Polk RE , Fox C , Mahoney A , et al. Measurement of adult antibacterial drug use in 130 US hospitals: comparison of defined daily dose and days of therapy. Clin Infect Dis 2007. 44:664–670. 17278056 10.1086/511640

[8] Morris AM. Antimicrobial stewardship program: appropriate measures and metrics to study their impact. Cur Treat Options Infect Dis 2014;6:101–112. 10.1007/s40506-014-0015-3 PMC4431704 25999798

[9] Al-Hasan MN , Winders HR , Bookstaver PB , et al. Direct measurement of performance: a new era in antimicrobial stewardship. Antibiot (Basel) 2019;8:127. 10.3390/antibiotics8030127 PMC6784134 31450576

[10] Winders HR , Al-Hasan MN , Jones BM , et al. Novel method of calculating adjusted antibiotic use by microbiological burden. Infect Control Hosp Epidemiol 2021;42:688–693 33504376 10.1017/ice.2020.1285

[11] Kadri SS , Adjemian J , lai YL , et al. Difficult-to-treat resistance in gram-negative bacteremia at 173 US hospitals: retrospective cohort analysis of prevalence, predictors, and outcome of resistance to all first-line agents. Clin Infect Dis 2018;67:1803–1814. 30052813 10.1093/cid/ciy378 PMC6260171

[12] Kadri SS , Lai YLE , Ricotta EE , Strich JR , et al. External validation of difficult-to-treat resistance prevalence and mortality risk in gram-negative bloodstream infection using electronic health record data from 140 US hospitals. Open Forum Infect Dis 2019;6:ofz110. 31240236 10.1093/ofid/ofz110 PMC6441782

[13] Tamma PD , Aitken SL , Bonomo RA , et al. Infectious Diseases Society of America 2023 guidance on the treatment of antimicrobial resistance gram-negative infections. Clin Infect Dis 2023 ;ciad428. doi: 10.1093/cid/ciad428. Online ahead of print. 37463564

[14] CDC. Antimicrobial Use and Resistance (AUR) Module. Atlanta, GA: U.S. Department of Health and Human Services, CDC; 2023.

[15] Al-Hasan M. Gram-negative bacteria with difficult-to-treat resistance: a moving target. Clin Infect Dis 2021;72:2121–2123. 32249916 10.1093/cid/ciaa384

